# Latest Management of Cardiac Sarcoidosis: A Comprehensive Review

**DOI:** 10.1155/crp/4913082

**Published:** 2026-06-04

**Authors:** Dunia Alhareth, Alaaeddine El Ghazawi, Sara Ajami, Zyad Saifi, Marwan Refaat

**Affiliations:** ^1^ Department of Internal Medicine, Cardiology Division, American University of Beirut Medical Center, Beirut, Lebanon, aubmc.org.lb; ^2^ Faculty of Medicine, American University of Beirut Medical Center, Beirut, Lebanon, aubmc.org.lb; ^3^ Department of Biochemistry and Molecular Genetics, American University of Beirut Faculty of Medicine, Beirut, Lebanon, aub.edu.lb

**Keywords:** cardiac sarcoidosis, device-based interventions, lifestyle modifications, pharmacological treatment

## Abstract

Cardiac sarcoidosis (CS) is a rare but serious manifestation of sarcoidosis. Its complications might include high‐grade conduction blocks, atrial and ventricular arrhythmias, and even heart failure. Despite its clinical significance, it exhibits a heterogeneous presentation that makes its diagnosis and management challenging. In this review, we aim to address the different management options of CS, encompassing pharmacotherapy, implantable devices, lifestyle modifications, and surgical interventions. Research findings suggested a positive role of corticosteroids in alleviating the inflammation associated with CS while also improving cardiac parameters. However, given their serious side effects, the use of steroid‐sparing medications was also supported. In addition, device‐based interventions and ablation were effective in managing the increased risk of arrhythmias and heart failure–associated CS. The importance of lifestyle modifications was tackled as a simultaneous treatment option along with the above. Still, interindividual variability and the absence of strict guidelines were major challenges.

## 1. Introduction

Cardiac sarcoidosis (CS) is a rare but severe manifestation of sarcoidosis, a multisystem granulomatous inflammatory disease most commonly affecting the lungs and lymph nodes. Cardiac involvement, although less frequent, carries significant clinical consequences, including arrhythmias, heart failure, and sudden cardiac death (SCD). [1]. Two distinct phenotypes of CS are recognized. Isolated CS involves granulomatous infiltration confined to the myocardium without extracardiac disease [2]. For this, diagnosis is particularly difficult, as extracardiac biopsy is not feasible, necessitating reliance on multimodality imaging. Systemic sarcoidosis with cardiac involvement, by contrast, presents with extracardiac manifestations such as pulmonary or lymph node disease, with cardiac involvement confirmed through complementary imaging and clinical assessment [3]. Although 2%–5% of patients with systemic sarcoidosis clinically have cardiac involvement, autopsy series show as many as 25% of cases with subclinical cardiac involvement [1, 4]. However, advances in diagnostic modalities such as cardiac MRI and positron emission tomography (PET) have enabled increased detection of subclinical disease [5].

Prognostic differences between the two are clinically significant. Isolated CS is associated with poorer outcomes, including reduced responsiveness to immunosuppressive therapy and higher rates of adverse cardiac events. In contrast, systemic sarcoidosis with cardiac involvement often benefits from earlier recognition via extracardiac biopsy and initiation of treatment during active inflammation, resulting in more favorable therapeutic responses [3]. This difference in terms of prognosis is likely due to the delayed diagnosis in the absence of extracardiac manifestations in cases of isolated CS in addition to the life‐threatening nature of cardiac symptoms. Furthermore, isolated CS is often associated with lower left ventricular ejection fraction (LVEF) and thus poorer survival [6].

Despite its clinical importance, the diagnosis and management of CS remain difficult because of its heterogeneous presentation, overlapping symptoms with other cardiac diseases, and the lack of a uniform diagnostic criterion [7]. Recent advances in imaging techniques and treatment modalities have improved our understanding of CS and its management; however, gaps in evidence and standardized guidelines still exist. Table 1 summarizes the current guideline’s diagnostic criteria and imaging modalities for CS with the corresponding advantages and limitations.

**TABLE 1:** Diagnostic criteria and imaging modalities in cardiac sarcoidosis with advantages and limitations.

Guideline	Components/criteria	Imaging role	Advantages	Limitations
HRS 2014 [8, 9]	Histologic or clinical pathways: extracardiac sarcoidosis + cardiac findings	CMR and PET mentioned but not formal major criteria	Structured, reproducible framework	Low sensitivity if biopsy negative; narrow scope, underuse of imaging
JCS 2017 [2, 9]	Expanded to include PET/CMR as major criteria; allows probable/possible CS	Imaging central to diagnosis	Higher sensitivity with PET/CMR practical in the real world	Risk of overdiagnosis, variability in interpretation, and imaging access limits
AHA 2024 [9–13]	Multimodal algorithm: clinical + imaging + electrophysiology; early detection emphasized	CMR, FDG‐PET central; combined PET/CMR discussed	CMR: fibrosis detection, prognostic value PET: sensitive for inflammation, therapy monitoring PET/CMR: improved accuracy	CMR: limited for distinguishing inflammation vs scar PET: preparation requirements, false positives PET/CMR: limited availability, not standardized

*Note:* FDG = fluorodeoxyglucose; EP = electrophysiology; MR/PET (or PET/CMR) = hybrid magnetic resonance positron emission tomography.

Abbreviations: AHA = American Heart Association; CMR = cardiac magnetic resonance; CS = cardiac sarcoidosis; HRS = Heart Rhythm Society; JCS = Japanese Circulation Society; PET = positron emission tomography.

CS’s manifestation ranges from asymptomatic left ventricular dysfunction to life‐threatening arrhythmias and cardiomyopathy. The three most common arrhythmias related to CS are atrioventricular block (AVB), followed by ventricular arrhythmias and atrial arrhythmias [14], which adds up to the high morbidity and mortality already associated with the condition. When left untreated, CS can progress to the development of chronic heart failure, necessitating heart transplantation (HTx) in extreme cases. The variability in the clinical presentation is a feature that usually delays diagnosis and even might be associated with poorer outcomes [15].

The management of CS is equally complex and involves a multidisciplinary approach in the vigorous integration of pharmacologic therapy (corticosteroids, anti‐inflammatory, and heart failure medications) and device implantation, along with lifestyle modifications. These different intervention strategies will be tackled as follows.

## 2. Methodology

For this comprehensive review on the management of CS, a systematic literature search using PubMed and MEDLINE was performed. Search terms included “sarcoidosis,” “cardiac sarcoidosis,” “management,” “treatment,” “steroids,” “immunosuppressive therapy,” “steroid‐sparing,” “antiarrhythmic,” “device therapy,” “ICD,” “pacemaker,” “lifestyle modifications,” and “guidelines.” Studies were limited to those published between 2005 and 2025 while also tackling CS or systemic sarcoidosis and reported outcomes attributed to pharmacologic therapy, device therapy, lifestyle interventions, or current guideline recommendations.

## 3. Pharmacological Treatment of CS

### 3.1. Corticosteroids

CS is an infiltrative disease characterized by the formation of noncaseating granulomas and inflammation. Given its inflammatory behavior, corticosteroids are often considered an effective first‐line therapy [16]. For instance, such therapy has been associated with a reduction in long‐term outcomes such as all‐cause mortality, heart failure–related hospitalization, and symptomatic arrhythmias specifically in patients with CS but with preserved LVEF and not in patients with moderately or severely impaired EF [17]. Furthermore, Scuppa and Accietto [18] noted that delayed or late treatment with immunotherapy might not reduce arrhythmic burden or improve cardiac parameters, thus leaving heart transplant as the only viable long‐term option. Delaying the treatment in this scenario may allow cardiac complications to worsen including parameters such as LVEF. Thus, treatment could become less effective, which might support the finding by Segawa and Taniguchi [17] by which steroids might not help when reaching the stage of moderately or severely reduced LVEF. Another study reported the effects of corticosteroids, mostly prednisolone [PSL], in addition to a few other drugs with an immunosuppressive effect, such as methotrexate (MTX), azathioprine, tacrolimus, and mycophenolate mofetil, on patients with isolated or systemic CS. In all the different drug combinations, patients with isolated CS showed a worse cardiac prognosis and less alleviation in disease activity as compared to patients with systemic sarcoidosis involving the heart [19]. The above results suggest that although corticosteroid might be an effective anti‐inflammatory agent in cases of CS, its use might be limited to a specific population of patients and to a specific period of time within the disease progression. In a survey by De Bortoli and Culver [20] in which 62 clinicians involved in CS treatment participated in answering a questionnaire related to CS therapy, prednisone, through its wide inhibitory effect on immune response, was considered a first‐choice agent in CS management. Still, challenges attributed to the dose of prednisone that should be administered and the tolerability of higher doses, in addition to the use of monotherapy or combination therapy, have been proposed by clinicians. For instance, 69.4% of the clinicians supported the use of prednisone monotherapy as a first‐line agent in CS management, whereas 25.8% suggested the use of a combination of prednisone with MTX [20]. In another study by Nagai and Yokomatsu [21], 17 patients with CS were treated with either combination therapy consisting of MTX and low‐dose corticosteroids (PSL) or corticosteroids alone. Interestingly, cardiac parameters including EF, left ventricular end‐diastolic diameter (LVDd), serum N‐terminal fragment pro‐brain natriuretic peptide (NT‐proBNP), and cardiothoracic ratio (CTR) all appeared to be more stabilized in the combination therapy group as opposed to the patients receiving corticosteroids only. This suggests an added benefit of the combination therapy compared to corticosteroids alone.

In conclusion, corticosteroids appear to be effective in the treatment of CS through reducing the risk of arrhythmias, most notably AV blocks, heart failure, and heart failure–related hospitalizations, thus mitigating the overall mortality risk. These beneficial effects, however, are most pronounced when the disease is detected early and corticosteroid therapy is initiated promptly. Furthermore, several limitations and challenges regarding its use still exist including its limited effect in cases of systemic sarcoidosis, knowing the optimal duration of therapy, understanding the importance of second‐line treatment therapies in some cases, and reaching the diagnosis early, as earlier detection is associated with better outcomes. As for the duration of therapy, there has been no clear consensus in the literature. Skowasch and Gaertner [22] reported that it is recommended to start with 30–60 mg of prednisone/day followed by a dose reduction to reach an initial maintenance dose equivalent to 10–15 mg for at least 6 months. This is followed by further dose reduction at 3–6‐month intervals with a total duration of therapy being at least 24 months. The clarified dose reduction is only done if pathological findings decline or if arrhythmias and left ventricular dysfunction do not exacerbate over time [22]. This suggests no set time frame for the therapy; it varies based on each patient’s progress.

### 3.2. Steroid‐Sparing Medications

Given their potential side effects and their long‐term serious consequences, some patients refuse the use of steroids [16]. Indeed, other therapeutic steroid‐sparing medications such as MTX have been suggested to be used in combination with corticosteroids, thus allowing the reduction in the dose of corticosteroid administered to the patient, along with their added conjugate benefits [16]. For instance, when CS was treated with MTX together with a low dose of PSL, there was amelioration of cardiac inflammation as compared to the patients receiving PSL only [23]. This might suggest a beneficial but not superior impact of MTX as compared to PSL. However, given that PSL might interfere with glucose levels, MTX might be considered the treatment of choice in diabetic patients having CS [23].

Furthermore, in a review by Palvia and Kaur [24] including 11 articles related to CS treatment, immunosuppressive agents such as MTX, mycophenolate, and azathioprine were considered to avoid the long‐term use of corticosteroids especially that they are capable of reducing inflammation. This conclusion is coherent with the previously mentioned results concerning the avoidance of steroid side effects. Interestingly, the 2025 PREDMETH randomized trial showed that MTX is noninferior to prednisone as a first‐line therapy in pulmonary sarcoidosis, with similar improvements in lung function at 24 weeks, supporting its role as an effective steroid‐sparing immunosuppressive agent that has also been extrapolated to the management of CS [25].

Besides, specific anti‐inflammatory agents such as infliximab and adalimumab, which target TNF alpha, in addition to rituximab, which targets B cells, could also be used in the management of refractory CS cases [24]. Ahmed and Okafor [26] further supported the use of TNF‐alpha inhibitor, as it is associated with a positive effect on atria–ventricular block recovery and left ventricular function. However, a major concern was the risk of infections associated with TNF alpha, which might require individualized antibiotic therapy [26]. Furthermore, the evidence supporting the use of TNF‐alpha inhibitors in CS is based mainly on observational studies and case series rather than RCTs. Consequently, efficacy and safety cannot be definitively established.

Hope and Chrusciel [27] aimed to compare the long‐term outcomes of patients with CS receiving steroids, disease‐modifying antirheumatic drugs (DMARDs), TNF‐alpha inhibitors, or no treatment at all. DMARDs and TNF‐alpha inhibitors were able to reduce the incidence of heart failure when compared to patients receiving either steroids or no treatment at all. However, the need for intracardiac device placement was not different between the groups [27]. Thus, it could be inferred that in addition to avoiding the side effects of steroids, steroid‐sparing medications might also have clinical importance. However, it is worth noting that among DMARDs, MTX usage might be preferred over mycophenolate mofetil and azathioprine, which could increase the risk of infection [27].

Other medications that might help manage sarcoidosis include abatacept, which modulates T‐cell activity, thus providing a benefit in inflammatory diseases including rheumatoid arthritis, psoriatic arthritis, and CS [28]. Abatacept seems to be cardioprotective and safe in terms of liver and bone marrow side effects when compared to MTX, azathioprine, or mycophenolate [28].

In a nutshell, although steroids alone might be indispensable in managing inflammation, several studies revealed a standalone effect of steroid‐sparing medications or a combined benefit resulting from their combination with corticosteroids. This allows tapering the doses of corticosteroids used and thus avoids their side effects. However, it is worth noting that although many of these steroid‐sparing drugs have desired clinical benefits, their side effects must be approached on an individual basis especially when it comes to the risk of infections as discussed previously. Table 2 summarizes the steroid‐sparing agents used in CS with their indications, effectiveness, risks, and sequencing.

**TABLE 2:** Steroid‐sparing agents in cardiac sarcoidosis: indications, effectiveness, risks, and sequencing.

Agent	Indications	Effectiveness	Risks/sequencing
Methotrexate (MTX) [29, 30]	Preferred DMARD when steroid sparing is needed	Reduces PET inflammation and allows steroid taper; lower infection risk vs AZA/MMF	Monitor liver, CBC; folate. Sequence: add early with steroids, escalate if inadequate
Mycophenolate mofetil (MMF) [29]	Alternative if MTX contraindicated or not tolerated	Similar outcomes vs MTX but higher infection risk; less robust PET/EF effects	GI, cytopenias, teratogenic. Second line; monitor infections
Azathioprine (AZA) [29]	Option when MTX/MMF unsuitable	Limited data; outcomes comparable but weaker PET/EF evidence	Myelosuppression, infection. Third‐line antimetabolite
TNF‐alpha inhibitors (infliximab, adalimumab) [27, 31]	Refractory/high‐burden disease with persistent PET activity or low EF despite steroids + DMARDs	Improve EF, reduce prednisone dose, suppress PET uptake	Infection risk; screen TB/HBV; caution in advanced HF. Escalate after steroids + MTX
Rituximab [32]	For refractory cases or intolerance to TNFi/antimetabolites	Small series: reduced PET uptake, symptom improvement	Monitor for infections, hypogammaglobulinemia
Abatacept [28, 33]	Consider in multirefractory or when TNFi contraindicated	Limited case evidence; potential benefit with favorable safety	Infections; slower onset. Rescue option

*Note:* MTX = methotrexate; MMF = mycophenolate mofetil; AZA = azathioprine; DMARD = disease‐modifying antirheumatic drug; TB = tuberculosis.

Abbreviations: CBC = complete blood count; EF = ejection fraction; HBV = hepatitis B virus; HF = heart failure; PET = positron emission tomography; TNFi = tumor necrosis factor‐alpha inhibitor.

### 3.3. Heart Failure and Antiarrhythmic Medications

When treating CS complicated with heart failure, the use of beta‐blockers might be recommended [34]. However, although beta‐blockers could be beneficial in heart failure management, they might exacerbate AVB, which is a possible complication of CS [35]. Thus, the use of beta‐blockers in CS patients requires caution. Moreover, Masunaga and Hashimoto [19] suggested the absence of beneficial cardiac outcomes of beta‐blockers and antiarrhythmics used in patients with isolated CS. Conversely, Segawa and Taniguchi [17] linked the improvement in EF in CS patients with impaired LVEF to “guideline‐directed medical therapy (GDMT)” such as angiotensin‐converting enzyme inhibitors (ACEi)/angiotensin receptor blockers (ARBs), beta‐blockers, and mineral corticoid receptor antagonists (MRAs). Furthermore, Palvia and Kaur [24] suggested the use of ARBs, aldosterone antagonists, and diuretics to improve left ventricular dysfunction triggered by CS.

The abovementioned findings suggest no clear approach for managing heart failure and cardiac dysfunctions in cases of CS. Although traditional heart failure therapies might be used in some patients with CS, these medications might have effects that coincide with the complications of CS, thus worsening the condition.

Currently, with SGLT2 inhibitors as pillars of modern GDMT, a multinational propensity‐matched analysis of patients with CS and HF by Ibrahim et al. [36] showed that SGLT2i use was associated with significant reductions in all‐cause mortality and hospitalizations. This suggests a potential benefit of SGLT2i in this infiltrative cardiomyopathy and thus possibly provides a better approach for managing HF in CS patients.

## 4. Device‐Based Interventions for CS

CS, being a granulomatous inflammatory disorder affecting the myocardium, often leads to complications such as ventricular arrhythmia, heart block, and heart failure. Device‐based interventions are used to manage such complications and conduction disturbances, especially in the presence of the risk of SCD [37]. Managing these complications frequently involves device‐based interventions, including implantable cardioverter–defibrillators (ICDs), pacemakers, cardiac resynchronization therapy (CRT), and mechanical circulatory support or heart transplants.

### 4.1. ICDs

ICDs are fundamental in preventing SCD in high‐risk CS patients, especially those with prolonged ventricular arrhythmias or a markedly decreased LVEF (< 35%) [38]. Nonetheless, risk stratification continues to be difficult, as studies demonstrate that patients with preserved LVEF may still encounter considerable arrhythmic risks, particularly in the presence of late gadolinium enhancement (LGE) on MRI, an established predictor of arrhythmic events [14]. A meta‐analysis encompassing 13 studies involving 1318 patients revealed that individuals with LGE exhibited a 20‐fold increased risk of experiencing ventricular tachycardia (VT) or SCD relative to those without LGE. The presence of biventricular LGE significantly increased the risk of ventricular arrhythmia [39]. Moreover, patients with AVB have a much greater likelihood for developing sustained VT, hence reinforcing the rationale for ICD implantation in these cases [40].

Emerging evidence indicates that CS patients with an LVEF of 36%–49% encounter a risk of ventricular arrhythmic events similar to those with an LVEF of < 35%, hence questioning traditional ICD eligibility criteria [41]. Recipients of secondary prevention ICDs have the greatest arrhythmic burden, indicating that risk evaluation must include parameters beyond LVEF, including previous VT/ventricular fibrillation (VF) episodes and myocardial scar burden. Recent research indicates that nonsustained VT (NSVT) and abnormal findings on 18F‐fluorodeoxyglucose (FDG) PET or gallium‐67 scintigraphy serve as independent predictors of fatal ventricular arrhythmias. This underscores the necessity for ICD assessment in CS patients, even those with mildly reduced LVEF [41]. Furthermore, an electrophysiology study (EPS) has been shown to be a useful risk stratification tool in CS, specifically in patients with intermediate LVEF and abnormal imaging, where EPS illustrated a positive predictive value of 100% and a negative predictive value of 93% for future ventricular arrhythmias even in patients with LVEF > 35% and no prior arrhythmia [42]. This shows that EPS can help identify patients who might benefit from ICD despite borderline EF.

The recent scientific statement from the American Heart Association (AHA) recommends a multimodal diagnostic strategy, asserting that ICD decisions must include PET and MRI results, clinical history, and genetic predisposition, rather than depending only on LVEF [10]. For instance, the AHA scientific statement identifies pathogenic variants in genes such as LMNA, DSP, FLNC, RBM20, and PLN as being associated with increased arrhythmic risk, which might influence ICD considerations and refine individualized risk stratification.

VT is a common and potentially fatal sign of isolated CS = , necessitating both prompt management and continuous risk evaluation. A case study by Puglla Sanchez and De Escalante Yanguela [43] highlighted sustained monomorphic VT as the primary manifestation of ICS, requiring synchronized cardioversion and subsequent ICD implantation for secondary prevention. Advanced imaging modalities, including cardiac MRI and PET‐CT, are essential for confirming myocardial inflammation and fibrosis, especially in individuals with VT suspected of having ICS. Due to the diagnostic complexities of ICS, it is essential to maintain a heightened clinical suspicion and prioritize early imaging evaluations to inform ICD choices and avert SCD.

Although ICDs have life‐saving potential, their use is not consistent across all patient groups, with notable gender disparities. Research indicates that although females experience higher rates of atrial fibrillation (AF) and VF, males are more likely to receive ICDs. Postimplantation outcomes are notably advantageous for females, with reduced occurrences of major adverse cardiovascular events (MACEs) and acute kidney injury (AKI) [44]. Although transvenous ICDs remain the standard, subcutaneous ICDs (S‐ICDs) offer an alternative, particularly for immunosuppressed patients at greater risk of vascular complications and infections. However, because S‐ICDs lack pacing capabilities, they are unsuitable for patients requiring bradycardia support [37]. Electrocardiographic indicators, including a prolonged T‐peak to T‐end (Tp‐e) interval, have been recognized as independent predictors of ventricular arrhythmic events, highlighting the need for careful risk assessment [45].

### 4.2. Pacemakers

Pacemakers are primarily indicated for CS patients with high‐degree AVB, a common early manifestation of the disease [14]. Although many AVB patients respond well to immunosuppressive medication, the high recurrence rate calls for permanent pacing, even in cases that initially appear transient [14]. Given the overlap between conduction disturbances and arrhythmic risk, many patients require a combination of ICD and pacemaker therapy, particularly those with persistent conduction abnormalities or unexplained syncope [38]. For instance, a study by Mkoko and Chin [46] indicated that cardiac implantable electronic devices (CIEDs), such as pacemakers and ICDs, were employed in 72.7% of CS patients, highlighting their essential function in addressing conduction abnormalities in the absence of advanced diagnostics. Moreover, it was indicated that patients with AVB and an LVEF ranging from 35% to 50% have a significant risk of SCD, warranting thorough evaluation of ICD therapy in conjunction with pacing [41].

### 4.3. CRT

Recent studies indicate that although CRT initially improves LVEF and cardiac remodeling in CS patients with heart failure with reduced EF (HFrEF), long‐term outcomes remain suboptimal. Patients with HFrEF demonstrate markedly poorer long‐term prognoses compared to those with mildly reduced EF (HFmrEF) or preserved EF (HFpEF), evidenced by elevated rates of mortality, transplantation, and hospitalization [47]. The findings underscore the necessity for more targeted treatment strategies within this population. Although CRT is an established therapy for HFrEF, its efficacy in CS varies. Although it may improve short‐term cardiac function, the long‐term advantages are often limited, especially for nonresponders like individuals with significant myocardial fibrosis, who tend to have worse outcomes [38].

A systematic review and meta‐analysis by Ahmed and Sivasankaran [48] analyzed 176 CS patients who received CRT and found that CRT produces short‐term benefits, such as increased LVEF and New York Heart Association (NYHA) functional class. Pooled data revealed an average LVEF increase of 3.75%, with a final pooled value of 34.28%. Despite these improvements, CS patients who received CRT had a significant prevalence of heart failure–related hospitalizations (23.2%) and major adverse cerebral and cardiovascular events (MACCE) (27%). Furthermore, only around 50% of CS patients responded successfully to CRT, a lower response rate than in other nonischemic cardiomyopathies, most likely due to the unique pathophysiology of CS. The study also said that for CS patients, CRT with a defibrillator (CRT‐D) is preferable to CRT with a pacemaker (CRT‐P) due to the increased risk of ventricular arrhythmias.

### 4.4. Mechanical Circulatory Support and Heart Transplantation

Left ventricular assist devices (LVADs) provide either a bridge to transplantation or long‐term support for patients with advanced heart failure, especially in cases of severe ventricular dysfunction that do not respond to medical therapy, including CS [24]. Furthermore, FDG‐PET has become a definitive tool in assessing active CS post‐LVAD implantation (18). In addition, in a 2025 cohort study by Okafor et al. [49], RV dysfunction and RV LGE on cardiac magnetic resonance (CMR) were present in a meaningful subset of patients with CS. This supports the need of appropriate RV evaluation through CMR or right heart catheterization in cases of any RV abnormalities detected on screening echocardiography.

As for HTx, although corticosteroids continue to remain as the foundational treatment for CS, HTx is the definitive treatment for end‐stage CS; however, strong immunosuppressive regimens and prolonged surveillance are necessary to prevent rejection and recurrence of the disease [50]. Indeed, CS patients treated with HTx appear to have either similar [51] or better short‐ and long‐term survival rates compared to HTx cases of other heart failure etiologies [50]. However, it is worth noting that in pooled data from reported cases in the literature, recurrence of CS in the transplanted heart was observed in about 5% of patients who underwent HTx [52]. This represents a further limitation of HTx in CS patients.

In conclusion, both HTx and LVADs are associated with superb outcomes in CS patients. Nonetheless, given the absence of standardized treatment guidelines, further research is needed to optimize timing and selection criteria for LVADs and HTx in CS patients.

## 5. Lifestyle Modifications

### 5.1. Diet and Nutrition

Dietary strategies have been increasingly acknowledged as a means to modulate inflammatory processes. Subsequently, obesity has been hypothesized to be a main contributor to the inflammation related to CA or a consequence of it. Obese individuals were found to have a 1.4–3.6× higher risk of developing sarcoidosis compared to people who were not obese [53]. Although this study does not specifically mention CS, it links obesity to sarcoidosis as a systemic disease that could involve the heart. The choice of the diet regimen followed by the individual will affect the course of disease onset and progression. The Mediterranean and DASH diets, which are rich in antioxidants, fibers, and omega‐3s, were found to significantly reduce C‐reactive protein levels and inflammatory markers in addition to providing a cardiovascular benefit. Given that CS is an inflammatory cardiovascular disease, this might suggest an indirect benefit of the Mediterranean diet in managing CS [54]. A high‐sugar diet might cause hyperglycemic peaks that induce inflammation and worsening of the symptoms. Patients with sarcoidosis are recommended to follow a low‐sugar diet to prevent exacerbation of systemic inflammation and their symptoms [55].

Around 8% of patients with sarcoidosis have hypercalcemia. Hypercalcemia is caused by the overproduction of 1,25‐dihydroxyvitamin D (calcitriol) by sarcoid granulomas [56]. Sarcoidosis patients taking vitamin D and calcium supplementation should be monitored closely as they are at increased risk of worsening hypercalcemia associated with sarcoidosis [57]. As CS typically occurs as part of systemic disease rather than in isolation, calcium and vitamin D supplementation may offer potential benefit.

### 5.2. Alcohol Consumption in CS

Alcohol consumption is usually seen more frequently in sarcoidosis patients with milder symptoms when compared to their counterparts with a more severe form of the disease [58]. This is explained by the more restrictive lifestyle choices in patients with a more advanced disease compared to the more flexible lifestyle of patients with a milder disease. However, alcohol consumption can induce oxidative stress, inflammatory responses, and direct myocardial toxicity, which could worsen the pre‐existing cardiac inflammation and fibrosis present in CS patients, leading to a higher risk of AF, myocardial infarction, and heart failure [59].

Although the above lifestyle modifications may benefit patients with CS given its inflammatory nature, direct evidence in this population is scarce and clinical decisions cannot be based solely on these findings.

## 6. Current Guidelines and Emerging Trends in Management

The current contemporary guidelines emphasize a multimodality, multidisciplinary approach to CS, yet notable differences remain across societies. The AHA (2024) highlights cardiac MRI and FDG‐PET as central diagnostic tools, with extracardiac histology preferred when feasible [10], whereas the JCS (2016) uniquely permits a clinical diagnosis of isolated disease using imaging and clinical criteria alone. More recently, the JCS/JHRS (2024) published a focused update on arrhythmia management in CS, highlighting the importance of refined risk stratification and updated device therapy recommendations [2, 60]. The ESC (2024) similarly advocates CMR and FDG‐PET for both diagnosis and disease monitoring [61], whereas the HRS (2014) framework remains more conservative, requiring extracardiac biopsy to support a clinical diagnosis in the absence of myocardial tissue [8]. All societies recommend corticosteroids as first‐line therapy; however, the AHA and ESC place greater emphasis on early introduction of steroid‐sparing agents such as MTX or mycophenolate, often guided by serial FDG‐PET. With regard to device therapy, the HRS and AHA/ACC/HRS guidelines provide Class I ICD indications for secondary prevention and for patients with reduced EF, whereas the AHA and ESC underscore ICD use in patients with high‐grade AVB, favoring it over a pacemaker alone [2, 8, 10, 61].

Although pharmacotherapy, device‐based interventions, and lifestyle modifications are considered the cornerstone of CS management, recent advancements have introduced additional effective methods for treating CS.

As discussed in a previous section, S‐ICD might represent a good alternative for CS management in patients who do not fit the criteria for implanting the traditional ICD. However, being associated with inappropriate shocks and the absence of pacing might restrict its use [37]. Knowing that patients with CS might have complications like AV blocks that require pacing suggests finding other appropriate ways of management. ElRefai, Menexi, and Roberts [37] mentioned that the use of leadless pacing (LP) is supported by the national expert consensus of the Austrian Society of Cardiology, the working group on LP of the Polish Cardiac Society, and the U.K. Expert Consensus Statement for the Optimal Use and Clinical Utility of Leadless Pacing Systems. For instance, Steinwender and Lercher [62] reported that LP can help decrease the possibility of infections and avoid lead‐associated complications while ensuring that pacing is present. However, it is worth noting that LP does not provide dual‐chamber pacing or antitachycardia pacing, which are needed for high‐grade AV blocks and VT management, respectively. These complications could be seen in CS, which would restrict its usage. Alternatively, in a study by Calvagna and Valsecchi [63], S‐ICD and LP were simultaneously implanted in 13 patients with CS, in which 11 had previously an infected ICD and 2 were planning to implant an ICD but had several risk factors for infection. This simultaneous implantation was effective, safe, and protective against infections while at the same time providing the features of an ICD and pacing therapy.

Another emerging therapy that has recently gained attention has to do with ablation. In a meta‐analysis by Adhaduk and Paudel [64], the role of catheter ablation in managing CS with refractory VT has been investigated. A total of 401 patients were studied. Acute procedural success was achieved in 57% of the patients; however, VT recurrence was equivalent to 55% after the first ablation and to 37% after several ablations. Adhaduk and Paudel [64] suggested that catheter ablation might be useful in patients with CS and refractory VT; however, a poor prognosis is expected in CS patients having refractory VT even after undergoing ablation. Furthermore, Al Taii and Saxena [65] reported that catheter ablation resulted in partial or complete success in 80% of patients studied. These patients presented with both VT and CS. Moreover, patients with preserved LVEF and absent active inflammation had better long‐term outcomes following the ablation [65]. Additionally, Palvia and Kaur [24] mentioned that although amiodarone and sotalol are initial treatments in CS cases complicated with VT, ablation is considered beneficial in refractory conditions or in cases of intolerance to antiarrhythmic medications. However, Arunachalam et al. mentioned that although ablation is recommended for refractory VT associated with CS, control of inflammation should precede the ablation. Furthermore, the outcome of ablation therapy is considered modest with a possibility of recurrence in 55% of the cases [14]. The above outcomes suggest a potential role of catheter ablation in the management of CS especially when complicated with refractory VTs as highlighted by the above studies. However, several challenges might arise using this approach. First, physicians must be able to detect the stage at which ablation becomes most effective, which should be after adequate control of the present inflammation. Additional challenges have to do with the high possibility of recurrence even after multiple ablations, in addition to the weak evidence supporting the ablation therapy given it stems mostly from observational studies rather than RCTs.

Moreover, the idea of personalized treatment, as previously introduced in the section related to pharmacotherapy, has to do with tailoring medical treatment according to each patient’s needs, complications, and progression. Qiao and Tian [66] also supported the idea of individualized therapy through advocating for the use of CMR and PET. Both techniques allow visualizing the different stages of CS, thus suggesting different personalized treatment plans [66]. Besides, Palvia and Kaur [24] reported that in cases of CS associated with heart failure, treatment should be personalized as “there is no one‐size‐fits‐all approach.” Thus, the use of personalized combination of traditional heart failure medications such as neurohormonal blockers and corticosteroids is recommended [24].

Finally, data comparing treatment efficacy across patients with isolated CS versus those with systemic involvement are poorly characterized. Table 3 summarizes the therapeutic responses discussed above in the subsequent patient groups along with the management implications.

**TABLE 3:** Clinical management in isolated cardiac sarcoidosis (ICS) vs systemic sarcoidosis with cardiac involvement (SSCI) and implications.

Management	ICS	SSCI	Management implication
Corticosteroids	Overall, less favorable response and prognosis than SSCI despite immunosuppression; late initiation may not reduce arrhythmias or improve cardiac parameters; benefit appears limited once moderate–severe LVEF impairment is present	First‐line anti‐inflammatory therapy; associated with reduced mortality, HF hospitalization, and symptomatic arrhythmias when LVEF is preserved and when treatment is initiated early	Prioritize early diagnosis and early steroid initiation. For dosing/tapering, one approach is to start prednisone 30–60 mg/day, reduce to 10–15 mg for ≥ 6 months, and then taper every 3–6 months; individualize based on the pathology/arrhythmias trajectory
Steroid‐sparing (DMARDs) (methotrexate, mycophenolate, azathioprine, tacrolimus, abatacept)	Often required early given relapse/side effects and poorer steroid response; in small cohorts, MTX + low‐dose PSL stabilized EF/LVDd/NT‐proBNP/CTR better than PSL alone	Used to reduce steroid dose and maintain control; reviews support MTX/MMF/AZA to reduce inflammation and prevent scarring; observational data suggest lower HF incidence vs steroids or no treatment	Favor PSL + MTX when feasible (consider MTX in diabetics); abatacept may be considered in select cases with a favorable cardiac safety profile relative to MTX/AZA/MMF
Steroid‐sparing (TNF‐α inhibitors) (infliximab, adalimumab; rituximab also used in refractory CS)	Consider for refractory ICS when inflammation persists despite PSL + DMARDs; reports of EF improvement and steroid‐sparing; monitor for infection risk	In refractory SSCI, associated with EF improvement, AV block recovery, and reduced steroid doses at 6–12 months; infection risk may influence DMARD choice (MTX often preferred over MMF/AZA)	Reserve TNF‐α inhibitors for refractory disease after PSL + DMARDs; individualize based on infection risk and comorbidities
Heart failure and antiarrhythmic medications	Limited benefit: poorer response to GDMT/antiarrhythmics reported in ICS; β‐blockers may exacerbate AV block—use with caution	In impaired LVEF, improvement in EF linked to HF therapies (ACEi/ARB, β‐blockers, MRAs); ARBs/aldosterone antagonists/diuretics suggested for LV dysfunction	Treat inflammation + substrate: Apply GDMT, but recognize ICS may be less reversible; be cautious with β‐blockers in conduction disease; tailor therapy to LVEF and rhythm
Device‐based interventions	High burden of VA, AV block, and HF; device therapy frequently needed to mitigate SCD risk; integrate MRI‐LGE/PET findings and clinical history for risk stratification	Similar indications guided by arrhythmic/conduction risk and imaging (LGE burden, PET activity); risk may persist even with preserved or mildly reduced LVEF when other risk markers are present	Use multimodal risk assessment (LGE extent—including biventricular LGE–PET activity, prior VT/VF, NSVT, AV block) rather than LVEF alone
Corticosteroids	Less effective if started late; limited benefit once severe LV dysfunction present	First line; improves symptoms, decreases mortality if started early	Start early; taper gradually (start prednisone 30–60 mg/day, reduce to 10–15 mg for ≥ 6 months, and then taper every 3–6 months)
Steroid‐sparing DMARDs (MTX, MMF, AZA, etc.)	Usually needed early due to relapse or steroid resistance	Reduce steroid dose and inflammation; prevent scarring	Combine with steroids (like PSL) when possible
Steroid‐sparing (TNF‐α inhibitors) (infliximab, adalimumab, rituximab also used in refractory CS)	Used in refractory disease	Improve EF and conduction issues in refractory cases	Reserve for steroid/DMARD failure + individualize therapy
Heart failure and antiarrhythmic medications	Limited response; β‐blockers may worsen AV block	Follow HF guidelines (ACEi, ARB, β‐blocker, etc.)	Treat inflammation + HF together
Device‐based interventions	Often required due to arrhythmia risk	Risk may persist even with preserved or mildly reduced LVEF when other risk markers are present	Use imaging + rhythm history for decisions

*Note:* AV block/AVB, atrioventricular block; CTR, cardiothoracic ratio; LVDd, left ventricular end‐diastolic diameter; MTX, methotrexate; MMF, mycophenolate mofetil; AZA, azathioprine; PSL, prednisolone; FDG, fluorodeoxyglucose.

Abbreviations: ACEi, angiotensin‐converting enzyme inhibitor; ARB, angiotensin II receptor blocker; CS, cardiac sarcoidosis; DMARDs, disease‐modifying antirheumatic drugs; EF, ejection fraction; GDMT, guideline‐directed medical therapy; HF, heart failure; ICS, isolated cardiac sarcoidosis; LGE, late gadolinium enhancement; LVEF, left ventricular ejection fraction; MRA, mineralocorticoid receptor antagonist; MRI, magnetic resonance imaging; NSVT, nonsustained ventricular tachycardia; NT‐proBNP, N‐terminal pro‐B‐type natriuretic peptide; PET, positron emission tomography; SCD, sudden cardiac death; SSCI, systemic sarcoidosis with cardiac involvement; TNF‐α, tumor necrosis factor alpha; VA, ventricular arrhythmias; VF, ventricular fibrillation; VT, ventricular tachycardia.

## 7. Implications for Clinical Practice

This review highlighted the beneficial role of corticosteroids in managing CS, which is well understood considering the inflammatory granulomatous nature of the disease. However, given the undesired side effects of corticosteroids, using steroid‐sparing medications, DMARDs, together with tapered doses of corticosteroids was also recommended. As CS progresses, the possibility of complications such as heart failure, arrhythmias, tachycardias, and AVBs is elevated. These complications were the reason behind considering heart failure and antiarrhythmic medications in addition to device‐based interventions such as ICDs, pacemakers, CRT, and LVADs. Although these manage the complications of the disease, still, they do not target the inflammatory behavior of CS. Furthermore, each advancement brought up new challenges attributed to different aspects such as disease progression, interindividual variability, and side effects. Besides, it is worth noting that in this review the effects of diet, socioeconomic status, and lifestyle modifications were adequately recognized as contributors to the overall CS management. However, in light of the different management approaches discussed, developing multidisciplinary treatment strategies that maximize benefits and minimize risks remains a major challenge. Venkataraj and Pierotti [28] mentioned that although recent treatment options promise 5‐year survival rates of 90%–96% and 10‐year survival of 80%–90%, unlocking the molecular–genetic pathogenesis through conducting large‐scale controlled trials might help further enhance treatment options and clinical outcomes. This might pave the way toward the desired tailored therapy for CS.

## Funding

No funding was obtained for this manuscript.

## Conflicts of Interest

The authors declare no conflicts of interest.

## Data Availability

Data sharing is not applicable to this article as no datasets were generated or analyzed during the current study.
